# Chronic Suppurative Lung Disease in Children: Definition and Spectrum of Disease

**DOI:** 10.3389/fped.2017.00030

**Published:** 2017-02-27

**Authors:** Gregory J. Redding, Edward R. Carter

**Affiliations:** ^1^Pulmonary Division, Seattle Children’s Hospital, University of Washington School of Medicine, Seattle, WA, USA; ^2^Pulmonary and Sleep Medicine, Banner Children’s Specialists, Banner Medical Group, Phoenix, AZ, USA

**Keywords:** bronchiectasis, bronchitis, abscess, suppurative, lung children, global

## Abstract

The most common clinical suppurative lung conditions in children are empyema, lung abscess, and bronchiectasis, and to a less often necrotizing pneumonia. Until recently, bronchiectasis was the most common form of persistent suppurative lung disease in children. Protracted bacterial bronchitis is a newly described chronic suppurative condition in children, which is less persistent but more common than bronchiectasis (1). In addition, the term “chronic suppurative lung disease” has been used recently to describe the clinical features of bronchiectasis when the radiographic features needed to make a diagnosis of bronchiectasis are absent. Webster’s New College Dictionary defines suppuration as the process of forming and/or discharging pus. Pus is a body fluid resulting from intense inflammation in response to infection that leads to neutrophil influx and apoptosis, microbial clearance, and often necrosis of nearby tissue. Pus is primarily composed of white blood cell debris.

## History

Pus has received written commentary since the first recorded medical works. Prescriptions for draining pus from abscesses were found in the Egyptian Medical Papyri (1550 BC) (2) and Hippocrates (430 BC) described empyema in several of his writings (3, 4). By the early 1800s, bronchiectasis, empyema, and lung abscess were recognized clinical entities associated with purulent secretions noted on autopsy but not yet linked to infection (5). In the latter half of the nineteenth century, the association of bacteria and infection with these entities evolved. In the 1870s, Pasteur reported that microorganisms were found in pus and were necessary to cause purulent infections (6). By the end of the nineteenth century, textbooks of bacteriology classified the “pyogenic bacteria” (*Staphylococcus aureus* and *Streptococcus pyogenes*), and noted that the most common cause of lobar pneumonia was pneumococcus (7). In the first quarter of the twentieth century, before the development of antibiotics, radiographic imaging became a standard diagnostic tool, which revolutionized the diagnosis of pneumonia, lung abscess, and pleural disease (8). In the 1800s, bronchiectasis was most often associated with sequelae of then common infectious diseases, including pertussis, measles, and tuberculosis, or due to the long-term effects of retained airway foreign bodies (9). Over the past 50 years, with the advent of vaccinations, improved nutrition, public health services, environmental awareness of indoor pollutants, and antibiotics/antifungal therapies, the prevalence of bronchiectasis has significantly decreased. It is now considered relatively uncommon in developed countries, though it still remains a significant cause of morbidity.

## Pathophysiology

The common themes of the suppurative pulmonary conditions are persistent and recurrent infection, intense neutrophilic inflammation, and poor clearance of infected material. Classic suppurative lung diseases are characterized by pus observed in expectorated sputum or sampled with drainage procedures through pleural catheters and with bronchoalveolar lavage.

Persistent inflammation becomes destructive but is often compartmentalized to a lung region. Lung abscesses are usually thick-walled localized regions; bronchiectasis, even in children with CF, occurs with only patchy peribronchiolar pneumonitis and could be considered an endobronchial abscess (10). Sepsis rarely occurs in children as a consequence of lung abscesses or bronchiectasis.

### Predisposing Factors

Poor pulmonary clearance of infected fluid is common; multiple mechanisms for this are listed in Table 1 along with some clinical examples. Recurrent aspiration in the presence of impaired host defense and/or tissue injury leads to recurrent and persistent airway infection. Poor oral hygiene and gingival disease contribute to this process in children with neurologic impairments and in children who aspirate following viral lower tract infections (11, 12). Children with postinfectious bronchiectasis have persistent colonization of their nasopharynx for years with the organisms isolated from expectorated sputum and bronchoalveolar lavage (13).

**Table 1:** **Mechanisms of poor clearance of infection and pus in children**.

External compression	Hilar adenopathy
	Tumors, cysts
	Vascular rings and slings
Endobronchial obstruction	Foreign body aspiration
	Endobronchial granuloma
Poor airway stability	Tracheo- and bronchomalacia (including TE fistula repair)
Congenital pulmonary airway malformations	Bronchial atresia, bronchial stenosis
	Congenital cystic adenomatoid malformation
	Intralobar sequestration, bronchogenic cyst
Micro-aspiration	Dysphagia syndromes
	Laryngeal cleft, TE fistula
Impaired cough	Neuromuscular weakness, impaired threshold to cough
Impaired cilia function	Cystic fibrosis (CF), ciliary dyskinesias
Impaired immune system	B-cell deficiencies (IgG deficiencies, IgG subclass deficiencies, IgA deficiencies), chronic granulomatous disease, combined variable immunodeficiency, and T-cell deficiencies
Abnormal mucus rheology	CF, ciliary dyskinesias
	Postinfectious bronchiectasis

Bronchiectasis is exacerbated by chronic exposure to environmental irritants and pollution. Tobacco and biomass (cooking fuel) smoke, ozone, and sulfur dioxide all increase goblet cell and submucosal gland mucus output (14). Sustained tobacco smoke exposure also increases goblet cell number and density in the airways, remodeling the airway into a hyper-secretory state. Bronchiectasis can also result from a “vicious cycle” of airway infection, as with lower respiratory tract viral infections, leading to airway injury, which is exacerbated by subsequent infections (15). Recurrent viral respiratory infections are more common with household crowding and daycare attendance and are associated with bronchiectasis in indigenous populations (16). Worldwide, malnutrition also plays a role by impairing host defenses. Of the immune deficiencies encountered in children presenting with bronchiectasis, B-cell disorders accounted for 73% of 131 children in a large case series (17). Of these, IgG deficiency accounted for two thirds of the cases. T-cell disorders (hyper-IgE syndrome, ataxia–telangiectasia, and Wiskott–Aldrich syndrome) accounted for only 7%.

### Airway Microbiology

The pathogens that produce suppurative lung disease in children include aerobic and anaerobic bacteria and, less frequently, fungi. Viruses may cause diffuse or patchy pneumonitis noted radiographically, but pus is rarely described. However, viruses such as adenovirus are known to cause bronchiolitis obliterans which in turn is complicated by bronchiectasis (18). The bacterial pathogens vary depending on the underlying specific clinical disorder (Table 2). Exceptions to this list include age-specific pathogens in the newborn period, e.g., Gram-negative organisms and group-B streptococcus. Fungal abscesses are more common in immunosuppressed patients, including those with oncological disorders and transplantation recipients. Children with immunodeficiencies, e.g., X-linked agammaglobulinemia and hyper-IgE syndrome, are predisposed to recurrent Staphylococcal infections, which can lead to lung abscess and chronic airway/parenchymal damage. Both aerobic and anaerobic bacteria have been isolated from lung abscesses in children (19). Among neurologically impaired children who underwent transtracheal aspiration to diagnose aspiration pneumonia, 67 of 74 (90%) aspirates grew both aerobic and anaerobic bacteria. *Aspergillus* infection complicates bronchiectasis in children with asthma and cystic fibrosis (CF) and can cause lung abscesses in immunocompromised patients.

**Table 2:** **Microorganisms isolated from patients with bronchiectasis**.

Cystic fibrosis	Chronic aspiration (11)	Postinfectious bronchiectasis (15)	Immotile cilia (20)	Lung abscess (21)
*Pseudomonas aeruginosa*	Alpha hemolytic streptococcus	*Haemophilus influenzae* non-type b	*H. influenzae* non-type b	Oral flora
*Staphylococcus aureus* (including MRSA)	*Staphylococcus aureus*	*Streptococcus pneumoniae*	*Staphylococcus aureus*	*Staphylococcus aureus*
*Stenotrophomonas maltophilia*	*Streptococcus pneumonia*	*Moraxella catarrhalis*	*Streptococcus pneumoniae*	*P. aeruginosa*
*Burkholderia cepacia* complex	*Peptococcus*	*Staphylococcus aureus*	*M. catarrhalis*	*Proteus mirabilis*
*H. influenzae* non-type b	*Peptostreptococcus*	*P. aeruginosa* (adults)		*Aspergillus* species
*Achromobacter*	*Fusobacterium*			Anaerobic organisms
Non-tuberculous mycobacteria	*Bacteroides melaninogenicus*			
	*Veillonella*			
	*P. aeruginosa*			

Of note, the presence of *Pseudomonas aeruginosa* isolated from airway cultures of children and adults with CF and adults with idiopathic bronchiectasis is associated with more rapid lung function decline and increased morbidity compared to those without Pseudomonal infection (22, 23). The age of initial isolation of *Pseudomonas* from sputum and/or bronchoalveolar lavage is much earlier in children with CF compared to children and young adults with postinfectious bronchiectasis and primary ciliary dyskinesia (PCD) (24–26). Among 68 infants with diagnosed CF undergoing surveillance bronchoalveolar lavage, 18% had *Pseudomonas* isolated by 2 years of age (26). In a separate prospectively studied cohort, 82 of 155 (53%) children with CF had *Pseudomonas* isolated by the time they reached 5 years of age (24). In contrast, 3 of 113 (2.7%) children 3 months to 16 years of age with newly diagnosed bronchiectasis unrelated to CF had >10^4^ CFU/ml of *Pseudomonas* isolated from bronchoalveolar lavage samples (27). Similarly, among 118 children 2–24 years of age with PCD, mucoid and non-mucoid *Pseudomonas* were recovered from 3 and 6%, respectively (20). *Pseudomonas* airway infection is often persistent in CF, PCD, and idiopathic bronchiectasis in adults and requires more aggressive and recurrent treatment than other bacterial pathogens.

### Airway Microbiota

Recent descriptions of lower airway microbiota in children and adults with CF- and non-CF-related bronchiectasis have provided new insights about these conditions. Characterizing populations of aerobic and anaerobic bacteria in the lung using culture-independent techniques such as nucleic acid sequencing, the lung is no longer considered a sterile site. New characteristics, such as diversity of lower airway bacterial populations, correlate directly with lung function and inversely with cough scores in adults with bronchiectasis (28). A decreasing diversity of bacterial populations in the lung is associated with more severe airway obstruction. Also, in a recent report comparing the lower airway microbiomes of 56 children 2–13 years of age with protracted bacterial bronchitis (PBB), bronchiectasis, and CF, the degree of diversity and the species membership of the core microbiota were remarkably similar across disease groups, the most common of which were shared with microbiota of sampled from normal children (29). The results differed from reports of microbiomes of adults with advanced airway disease suggesting the microbiome transitions in disease specific ways with time, treatment, and progression of disease.

## Specific Chronic Suppurative Conditions

Bronchiectasis is defined by the abnormal appearance of the conducting airways by high-resolution computerized tomography (HRCT) of the chest, producing dilation, thickened airway walls, and saccular changes in the clinical setting of chronic wet cough with or without expectoration of mucopurulent sputum. Bronchiectasis can be diagnosed in some chest radiographs, but this is an insensitive way to make the diagnosis and is not considered the gold standard. Bronchiectasis is the result of chronic airway infection leading to loss of structural integrity of the airway wall, which produces airway dilation. It can occur focally after serious acute respiratory infections or with post-obstructive pneumonia due to foreign body or endobronchial lesions (tumor and tuberculosis) or throughout the lung in a patchy distribution (e.g., CF and PCD). Bronchiectasis can also develop after severe lower respiratory viral infections, especially in indigenous populations (18, 30). Bronchiectasis results in airway dilation, described as cylindrical or saccular in shape, diagnosed by HRCT. The relatively increased size of the airway lumen compared to its neighboring pulmonary artery produces a “signet ring” appearance, diagnostic of bronchiectasis. However, the normal ratio of the airway to the vessel diameter is age dependent in normal children (31).

Protracted bacterial bronchitis is a recognized entity encountered primarily in young children. It is defined by a “wet” cough lasting longer than 1 month. It is likely a precursor to bronchiectasis in some cases, but it can be completely eliminated with two to three prolonged courses of antibiotic treatment (32). Children with PBB are often too young to expectorate sputum even though they present with a “wet” cough. Only through clinical studies using samples obtained by bronchoscopy with bronchoalveolar lavage do we know that this condition is characterized by neutrophil-rich sputum that is culture positive for bacterial pathogens (33).

Chronic suppurative lung disease (CSLD) is a broad descriptive term, but it has recently been specifically applied to children who clinically appear to have bronchiectasis but do not demonstrate the diagnostic imaging features of bronchiectasis. Children with CSLD respond to the same treatment regimens used for childhood bronchiectasis, and some authors consider them a spectrum of the same disorder. Of note, they differ from children with PBB in that their conditions cannot be completely reversed with multiple courses of antibiotics. In a 2-year prospective study, 43% of 161 children with PBB had >3 episodes of wet cough per year despite antibiotic treatment (32). The number of treatment failures that constitutes a change in diagnosis from PBB to CSLD has not been defined. Thirteen percent of children with PBB eventually demonstrated radiographic evidence of bronchiectasis (32). PBB and CSLD likely represent a continuum of chronic suppurative airway disease and recurrent treatment “failure” of children with PBB should raise the question of CSLD or bronchiectasis.

## Global Disparities in Suppurative Childhood Lung Disease

There remain glaring disparities among children in different societies who are at risk for CSLD. Access to vaccines for *Haemophilus* type b, pneumococci, and influenza, and comprehensive longitudinal health care vary significantly across and within countries. Among wealthy nations with effective public health infrastructures and proactive well-child care, suppurative lung disease is uncommon and usually occurs in the context of a known pulmonary or immune host defense disorder. However, among high-risk indigenous groups in Australia, New Zealand, Argentina, and Alaska, postinfectious bronchiectasis is most common. There is ongoing debate about the relative role of social and environmental risks versus immune deficiencies and abnormal lung injury and repair processes in different populations of children (16, 34, 35). It is of interest that a high rate of consanguinity has reported in several series of families whose children have idiopathic bronchiectasis, suggesting a genetic predisposition to bronchiectasis in some groups (36, 37).

**Figure 1:**
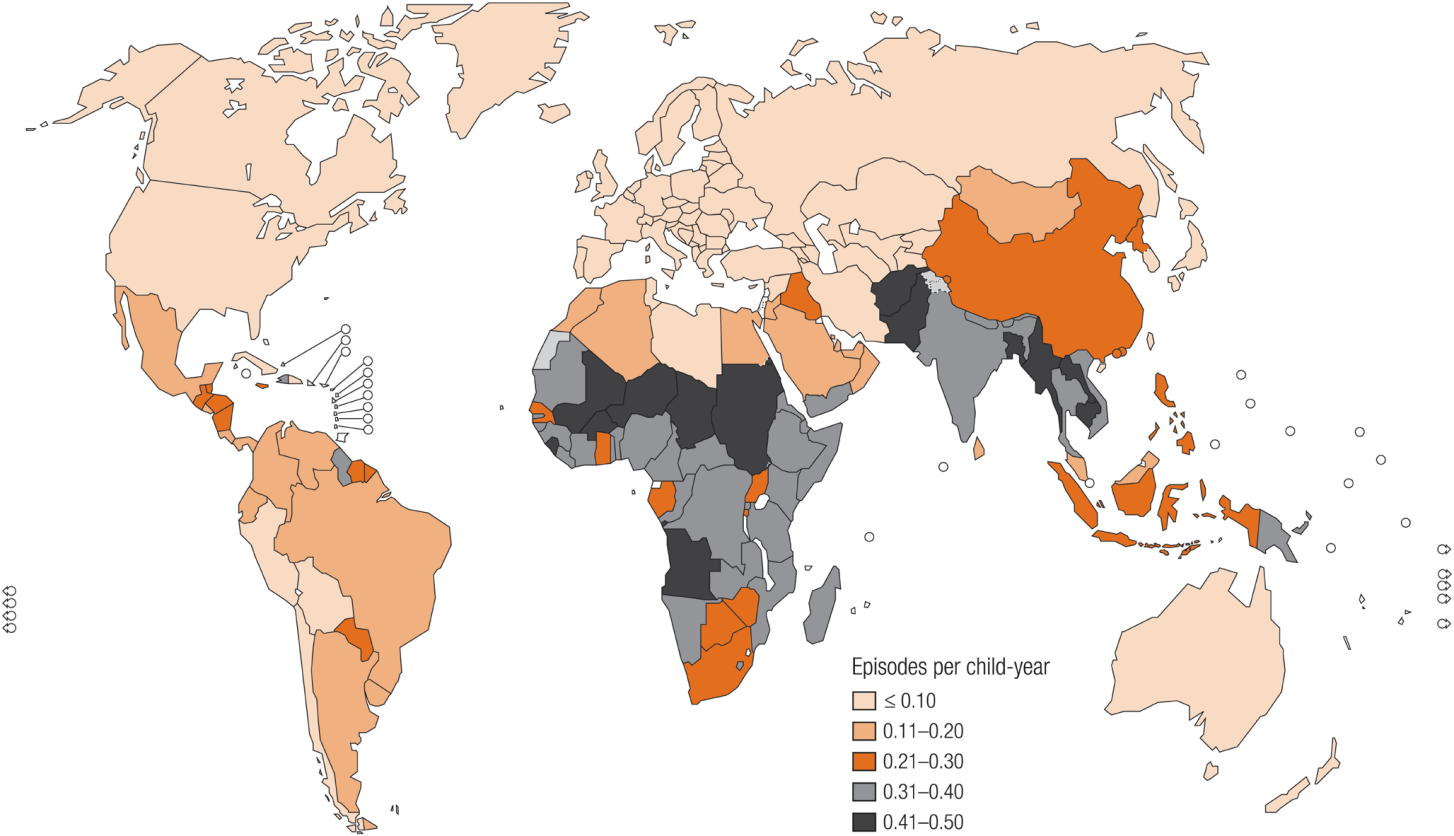
**Episodes of acute pneumonia per child year produced in 2007 by the Child Health Epidemiology Reference Group, established by the World Health Organization**. Reproduced with permission from Rudan et al. (38) and the World Health Organization.

**Figure 2:**
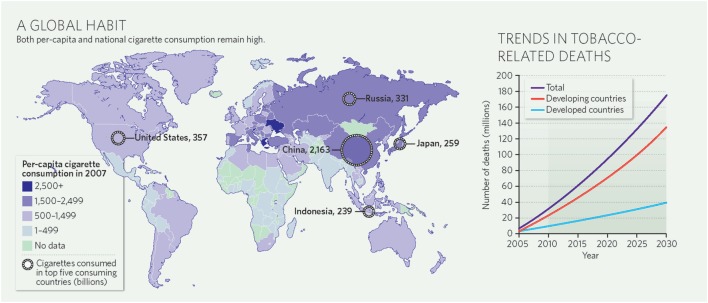
**Cigarette consumption (#cigarettes smoked per person per year among adults) compiled by the Tobacco Atlas in 2015**. Reproduced with permission from Copyright Clearance Center license #3981000761719.

**Figure 3:**
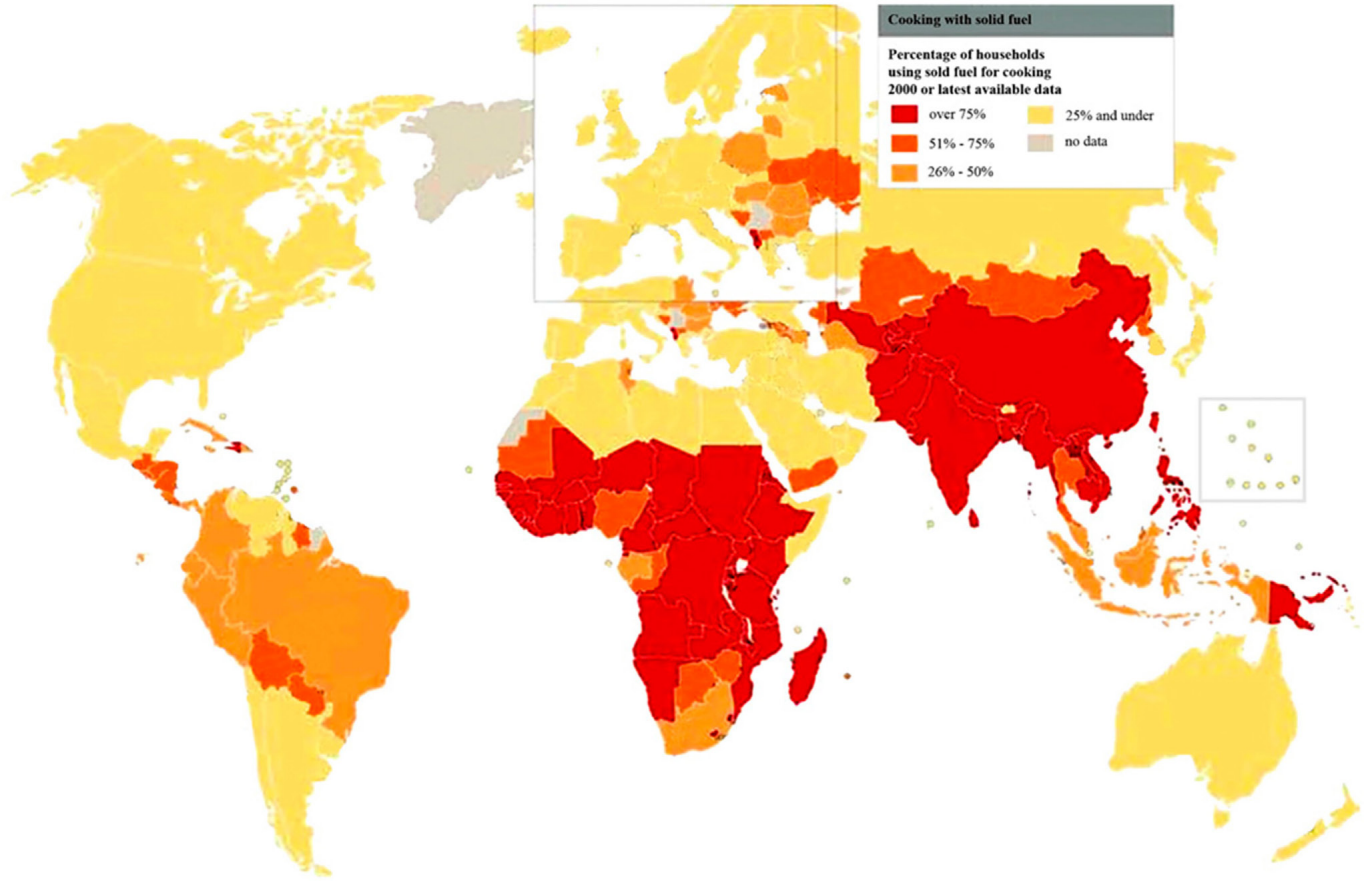
**Percentage of households using solid fuel for cooking in 2000 according to the World Health Organization**. Reprinted with permission of the American Thoracic Society. Reproduced from Copyright © 2016 American Thoracic Society. The *Proceedings of the American Thoracic Society* is an official journal of the American Thoracic Society. It was renamed the *Annals of the American Thoracic Society* in 2013 (40).

**Figure 4:**
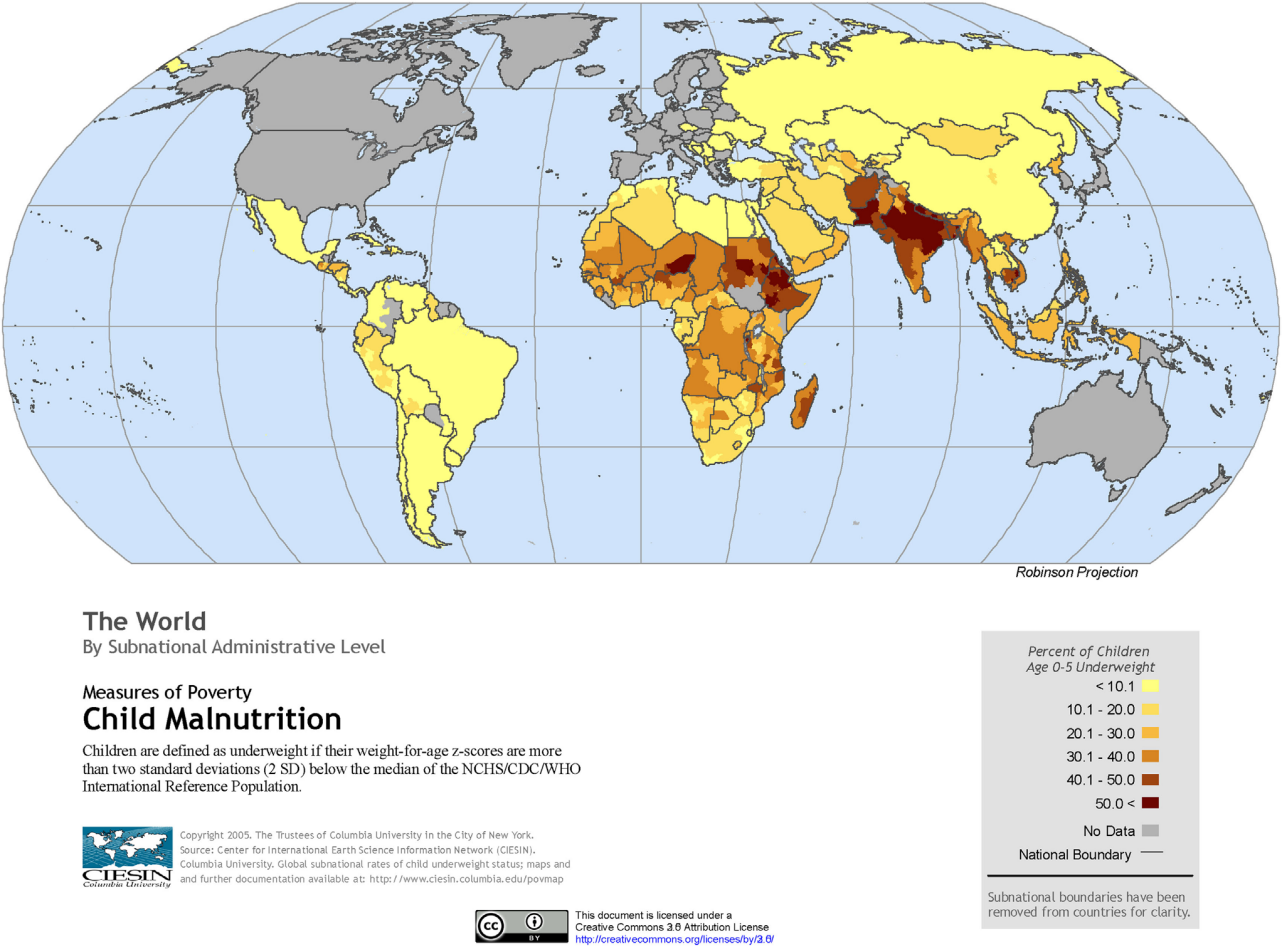
**Percentage of children <5 years old who are underweight produced by the Center of International Earth Science Information Network in 2005**. Reproduced with permission from Center for International Earth Science Information Network (CIESIN), The Earth Institute, Columbia University (41).

**Figure 5:**
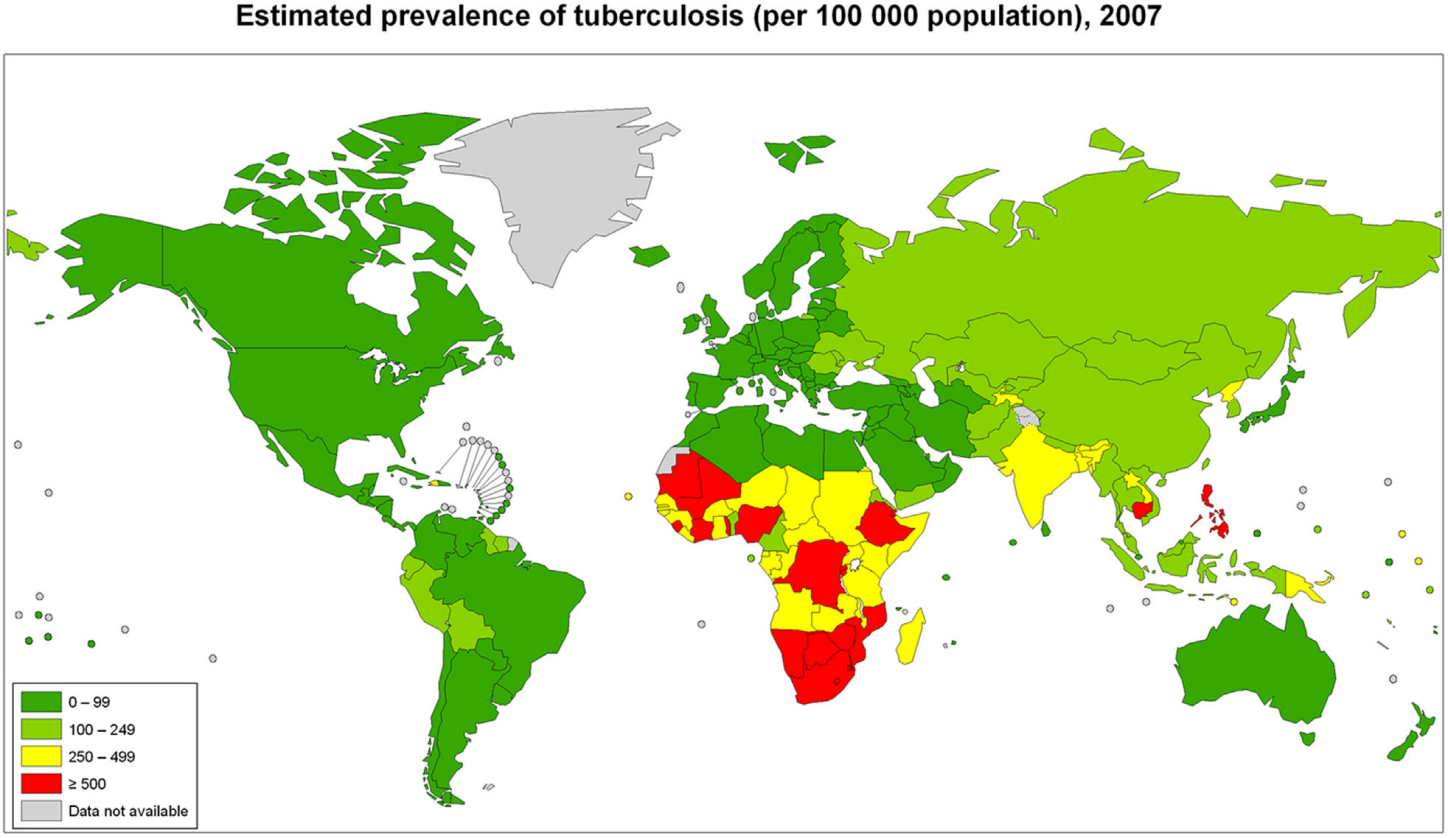
**Tuberculosis cases in adults and children per 100,000 populations in 2007 compiled by the World Health Organization**. Reproduced with permission from World Health Organization Map Production: Public Health Information and Geographic Information Systems (GIS) World Health Organization.

The global burden of CSLD in children is unknown. The geographic prevalences of bronchiectasis are limited due in part to lack of access to imaging technology need to make the diagnosis. Those regions where prevalences of acute pneumonia, tuberculosis, HIV infections, malnutrition, and tobacco and biomass combustion are highest are likely to be the regions where CSLD is more frequent. Figures 1–5 depict the variations in frequencies of risk factors across countries and geographic regions. The world map of episodes per child year of acute pneumonia reported in 2007 is depicted in Figure 1 (38). Figure 2 is a map of worldwide cigarette consumption (# cigarettes smoked per person per year for individuals >15 years of age) according to the Tobaccos Atlas in 2015 (39). The percentage of households using solid fuel (biomass) for cooking in 2000 is depicted in Figure 3 (40). Figure 4 illustrates the global percentage of children <5 years old in 2005 who are underweight, a reflection of malnutrition (41). If these maps are superimposed, the highest risk for CSLD in children would occur in Southeast Asia, China, and Sub-Saharan Africa. Prevalence data about persistent suppurative lung conditions in children in most of these areas have not been reported.

**Figure 6:**
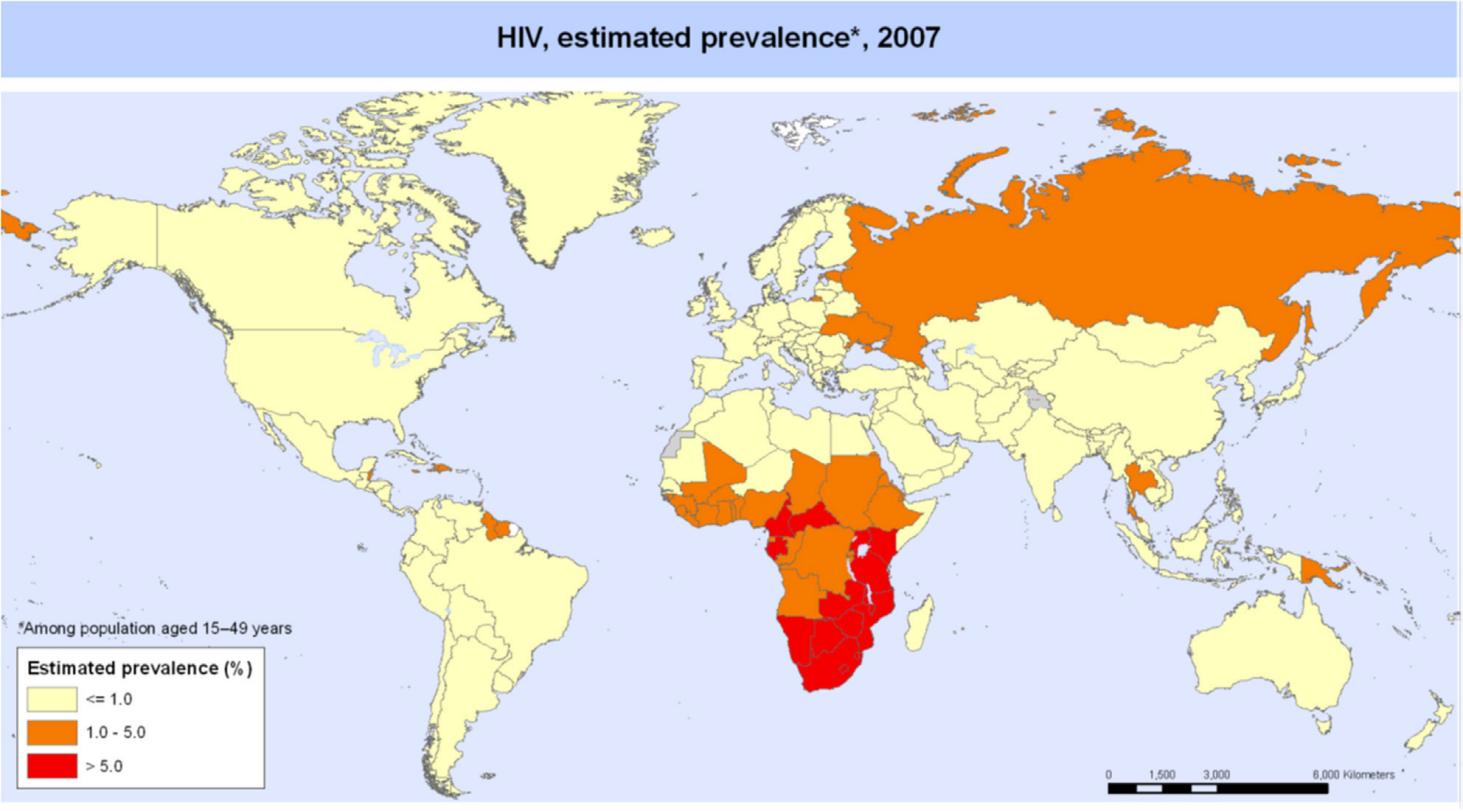
**HIV disease prevalence in 2007 compiled by the World Health Organization**. Reproduced with permission from WHO/UNAIDS Map Production: Public Health Information and Geographic Information Systems (GIS) World Health Organization.

Tuberculosis in children with and without HIV is also global concern. Several reports of chest CT findings in children with tuberculosis describe bronchiectasis occurring in 9–20% and cavitary lesions in 25% of adolescents (42, 43). The Global Tuberculosis Programme at the World Health Organization estimated that 1 million children developed tuberculous disease in 2014 (44). Seventy-five percent of children with tuberculosis come from just 22 countries. Of these, India alone accounts to 27% of the global burden of tuberculosis in children <15 years of age (45). The global frequency of tuberculosis cases per 100,000 population in 2007 is portrayed in Figure 5 (46). The map of HIV disease prevalences also compiled by the World Health Organization in 2007 is portrayed in Figure 6 (47). These maps suggest that bronchiectasis related to tuberculosis and HIV occurs most frequently in Africa, India, and parts of Southeast Asia.

## Long-Term Consequences of Suppurative Lung Disease

Most children with acute suppurative processes, e.g., pneumonia, empyema, abscess, if treated appropriately, make full recoveries with minimal long-term morbidity. However, apart from CF, the long-term consequences of CSLD in children have not been well described. Serial lung function trends for children with CF and non-CF postinfectious bronchiectasis have been reported (48). Non-CF bronchiectasis is associated with worse lung function early in life soon after the initial airway injury, but it progresses much more slowly over time compared to CF-related bronchiectasis. Complete resolution of symptoms among Native Alaskan children with postinfectious bronchiectasis occurs in one-third by adolescence (49). Although the mortality risk is greater in adults with bronchiectasis than among adults with asthma, it is lower compared to adults with COPD, at least in developed countries (50). Among adults with COPD, mortality is higher when it bronchiectasis is also present. Systems of care that transition youth and young adults with CSLD to adult providers have been best developed for patients with CF and, more recently, for those with ciliary disorders. Comprehensive care models of non-CF-related bronchiectasis have been developed that stabilize and/or improve lung function over time (51). However, the impact of smoking tobacco and other combustible products among adolescents and young adults with bronchiectasis has not been reported. Also, these models have not accounted for eventual transitions to adult care.

## Conclusion

With the advent of antibiotics, improved nutrition, and focus on child health in developed countries, bronchiectasis has been termed an “orphan disease.” Relative to the research devoted to CF, the published research on care of children with other chronic suppurative respiratory disease is scarce. Although the World Health Organization has prioritized asthma, acute pneumonia, and tobacco use, the burden of CSLD in children is unknown. Given the geographic distribution of risk factors worldwide, it is likely that children with these conditions are underdiagnosed and undertreated. The global burden of CSLD in children and its impact on health in adulthood are important to ascertain. Only then can effective systems of comprehensive longitudinal care in the context of different cultures be devised.

## Author Contributions

GR wrote entire article except history section. EC wrote history section and reorganized the article into current format.

## Conflict of Interest Statement

The authors declare that the research was conducted in the absence of any commercial or financial relationships that could be construed as a potential conflict of interest.

## Abbreviations

ABPA, allergic bronchopulmonary aspergillosis; CF, cystic fibrosis; CSLD, chronic suppurative lung disease; HRCT, high-resolution computerized tomography; PBB, protracted bacterial bronchitis; PCD, primary ciliary dyskinesia.
